# Effects of SW033291 on the myogenesis of muscle-derived stem cells and muscle regeneration

**DOI:** 10.1186/s13287-020-1574-5

**Published:** 2020-02-21

**Authors:** Yuanqiang Dong, Yuan Li, Chuan Zhang, Haibin Chen, Lijia Liu, Simeng Chen

**Affiliations:** Department of General Surgery, The First Affiliated Hospital of Nanjing Medical University, 300 Guangzhou Road, Nanjing, 210029 Jiangsu People’s Republic of China

**Keywords:** Muscle-derived stem cells, Small-molecule inhibitor, SW033291, Myogenic differentiation, Muscle regeneration

## Abstract

**Background:**

The unmet medical needs in repairing large muscle defects promote the development of tissue regeneration strategy. The use of bioactive molecules in combination with biomaterial scaffold has become an area of great interest. SW033291, a small-molecule inhibitor targeting 15-hydroxyprostaglandin dehydrogenase (15-PDGH) and subsequently elevating the production of prostaglandin E2 (PGE2), has been proved to accelerate the recovery and potentiate the regeneration of multiple tissues including the bone, liver, and colon. The limited understanding of the potential therapeutic effects on myogenesis motivated us to investigate the role of SW033291 in regulating muscle-derived stem cell (MDSC) myogenic differentiation and MDSC-mediated muscle regeneration.

**Methods:**

The characteristics of rat MDSCs, including cell-specific markers and myogenic differentiation potential, were determined. MDSCs were incubated with SW033291 to evaluate PGE2 production and cytotoxicity. The effects of SW033291 on MDSC myogenic differentiation were assessed by quantitative real-time polymerase chain reaction (qPCR), western blot, and immunocytochemistry. The fibrin gel containing MDSCs and SW033291 was used for muscle regeneration in a tibialis anterior muscle defect model.

**Results:**

Our data demonstrated that MDSCs were well-tolerated to SW033291 and treatment with SW033291 significantly promoted the production of PGE2 by MDSCs. In vitro analysis showed that SW033291 enhanced the myogenic differentiation and myotube formation by upregulating a series of myogenic markers. Additionally, the activation of PI3K/Akt pathway was involved in the mechanism underlying these promotive effects. Then, in situ casting of fibrin gel containing MDSCs and SW033291 was used to repair the tibialis anterior muscle defect; the addition of SW033291 significantly promoted myofiber formation within the defect region with mild immune response, less fibrosis, and sufficient vascularization.

**Conclusion:**

SW033291 acted as a positive regulator of MDSC myogenic differentiation, and incorporating the compound with MDSCs in fibrin gel could serve as an effective method to repair large skeletal muscle defects.

## Introduction

Skeletal muscle consists of myofibers, connective tissues, and an organized network of vascular and nerves, which is responsible for body movement and locomotion [1]. Skeletal muscle possesses regenerative potential following minor muscle damages, and local inflammation triggers the release of various biological factors and activates the signaling pathways leading to muscle stem cell recruitment, expansion, and differentiation, thus regenerate myofibers and repair the damaged tissue [2]. However, some traumatic incidents such as motor vehicle accidents, explosive injuries, and iatrogenic causes will lead to volumetric muscle loss (VML); in such cases, endogenous self-repair becomes severely impaired due to the insufficient muscle stem cell recruitment and loss of appropriate regenerative signaling, resulting in non-functional scar formation instead of myofiber stumps [3]. To date, the repair of volumetric muscle loss still remains a great challenge, as the current standard of care in clinic, engraftment of autologous muscle flaps, is usually limited by insufficient supply of muscle tissue and considerable donor site morbidity. Alternatives such as biological acellular scaffolds and minced muscle tissue have also been applied in repairing VML; however, all these techniques suffer from multiple disadvantages [4, 5]. There still exists an unmet medical need for the enhancement of functional muscle regeneration.

To achieve the goal of functional integration and recovery of damaged skeletal muscle, regenerative medicine represents a promising therapeutic strategy, which has involved the use of seed cells with myogenic origin such as satellite cell (SC), myoblast and muscle-derived stem cell (MDSC), or non-myogenic origin as mesenchymal stem cell (MSC) from bone marrow or adipose tissue [6]. Recently, considerable efforts have been made towards the augmentation of seed cell expansion and differentiation, including mechanical stimulation, electrical stimulation, and biochemical activation by growth factors and other biomolecules, and the underlying molecular mechanism governing this process has also been elucidated to some degree [7]. Among biochemical stimulation approaches, growth factors such as hepatocyte growth factor (HGF) and insulin-like growth factor (IGF) have been demonstrated to regulate muscle tissue homeostasis and regeneration by modulating the proliferation and differentiation of satellite cells and myoblasts [8, 9]. Other growth factors targeting the non-muscle components, including vascular endothelial growth factor (VEGF) and nerve growth factor (NGF), are also essential for developing a functional tissue [10–12]. These growth factors can be injected systematically or delivered to the damaged region in combination with bioactive scaffolds; however, there are still some limitations including complications of immunogenicity, short half-life, low bio-stability, and high manufacturing costs [13, 14]. As an alternative to direct growth factor delivery, genetic modification of seed cells using either viral or non-viral vectors enables in situ synthesis of growth factors and biomolecules of importance within the site of injured [15]. But from a safety perspective, viral vectors may induce immune reactions, which result in unexpected damaging results; although non-viral vectors such as liposomes and synthetic plasmid particles show less safety concern when compared to viruses, they have been initially associated with reduced gene modification efficiency [16, 17].

Another important category of bioactive molecules used for skeletal muscle tissue engineering is small molecules; they can be synthesized with controlled physicochemical properties, excellent permeability, and most importantly, high selectivity and potent bioactivity against target molecules, also with relatively lower manufacturing cost [18, 19]. The emergence of high-throughput screening technologies has enabled the discovery of small molecules that control cell behavior while also activating signaling pathways related to skeletal tissue regeneration [20, 21]. For example, retinoic acid signaling plays an essential role in regulating the differentiation of muscle progenitor cells; selective retinoic acid receptor-γ (RARγ) agonists have been demonstrated to promote repair of injured skeletal muscle in mouse [22]. Other potential small bioactive molecules for skeletal muscle regeneration include BIO (glycogen synthase-3 kinase inhibitor), SQ22536 (adenylyl cyclase inhibitor), SB203580, (p38-MAPK inhibitor), and lysophosphatidic acid (pleiotropic activator of G-protein-coupled receptors) [23]. As more and deeper insights into the regulatory role of small molecules presenting the benefits of delivering small molecules in supporting muscle regeneration become increasingly apparent, it is likely that the delivery of therapeutic small molecules will develop into a more widely used approach in muscle regeneration.

Prostaglandin E2 (PGE2), a lipid signaling molecule that supports expansion of several types of tissue stem cells, is a candidate therapeutic target for promoting tissue regeneration. PGE2 derives from arachidonic acid, which is released from membrane phospholipids by phospholipase A2 and sequentially produced by cyclooxygenase-1 and cyclooxygenase-2 enzymes (COX-1 and COX-2) [24]. PGE2 augments Wnt signaling, a pathway that is involved in the maintenance of several types of tissue stem cells, including hematopoietic and colon stem cells [25, 26]. In response to muscle injury, PGE2 is also synthesized and secreted by immune and myogenic cells, leading to significant activation and expansion of satellite cells and myoblasts through specific binding to e-type prostanoid receptor 4 (EP4 receptor) [27, 28]. We hypothesize that agents promoting PGE2 level could be beneficial in stimulating recovery of damaged muscle tissue. Potential strategy in promoting PGE2-mediated tissue regeneration could be either increasing PGE2 synthesis or inhibiting its rapid degradation. Recently, exciting data present that pharmacological inhibition of 15-hydroxyprostaglandin dehydrogenase (15-PGDH), which is a negative regulator of prostaglandin E2, using a small molecule inhibitor SW033291 significantly increases PGE2 levels in vivo and accelerates hematopoietic recovery in mice receiving a bone marrow transplant, and it also promotes tissue regeneration in mouse models of colon and liver injury [29]. Furthermore, another research has revealed that injection of SW033291 significantly boosts bone formation through the activation of PGE2/EP4 signaling [30]. Although inspiring data have shown SW033291 as promising therapeutic small molecule in the regeneration of various tissues, the direct effects of SW033291 on muscle regeneration have not been investigated and the underlying molecular mechanism is not fully understood.

In this study, muscle-derived stem cells (MDSCs) were chosen as the seed cells; these cells can be expanded exponentially in vitro, so there is no source limitation and have sustained proliferation, self-renewal, and differentiation potential [31, 32]. We systematically analyzed the effects of SW033291 on the myogenic differentiation of MDSCs and further investigated the underlying mechanism. Additionally, we evaluated the potential of SW033291 on MDSC-mediated muscle regeneration in a tibialis anterior muscle defect model. Our findings revealed the novel role of small-molecule inhibitor SW033291 in the regulation of MDSC-mediated muscle regeneration, suggesting that SW033291 may be a promising therapeutic agent in repairing muscle defect.

## Methods and materials

### Cell isolation and culture

All animal procedures were approved by the Institutional Animal Care and Use Committee of Nanjing Medical University. Muscle-derived stem cells (MDSCs) were isolated according to a previously reported protocol [33]. Briefly, muscle tissues were harvested from the hindlimb of 8-week-old female Sprague Dawley (SD) rats (Shanghai Animal Experimental Center, China). After mincing into small pieces, the tissues were treated with 0.1% collagenase, dispase, and trypsin solution (all from Sigma-Aldrich Corp., St. Louis, MO, USA) and incubated at 37 °C for 24 h. Dissociated cells were then processed in accordance with a reported serial pre-plating method using collagen-coated plates at 37 °C with a 5% CO_2_ humid atmosphere; passage 6 MDSCs were used for the experiments. For in vivo experiments, MDSCs were labeled by green fluorescent protein (GFP) using a lentivirus system purchased from GeneChem (GeneChem Technology Co., Ltd., China) according to the manufacturer’s instructions [34]. Flow cytometry was used to characterize MDSC surface marker using specific antibody against Sca-1, CD105, CD4, and CD45 (Invitrogen, Carlsbad, CA, USA) [33]. Growth medium (GM) for MDSCs was DMEM (Invitrogen) supplemented with 10% FBS (Invitrogen) and 100 units/ml of penicillin and streptomycin (Invitrogen). The differentiation medium (DM) was DMEM containing 2% horse serum (Invitrogen) and 100 units/ml of penicillin and streptomycin [35]. For SW033291 treatment, the small-molecule inhibitor was purchased from MedChemExpress LLC (Monmouth Junction, NJ, USA), then dissolved into dimethyl sulfoxide (DMSO) and incubated with MDSCs at different concentration ranging from 20 to 1000 nM. For PI3K/Akt signaling pathway inhibition, LY294002 (Thermo Fisher Scientific Inc., Waltham, MA, USA) was added into DM and GM at a final concentration of 50 μM according to previously reported protocol [36].

### Cytotoxicity and ELISA

To investigate the cytotoxicity of SW033291, MDSC cells were incubated with the compound at different concentrations in the range of 20 to 1000 nM and subsequently evaluated by senescence-associated β-galactosidase (SA β-Gal) staining, LIVE/DEAD staining (Invitrogen), and Alamar blue assay (Invitrogen) as previously described [35, 37]. SA β-Gal staining was performed with a cellular senescence assay kit (Millipore, Billerica, MA) according to the manufacturer’s instructions. Percentages of SA β-Gal-positive cells which show blue-green staining pattern were determined for each sample using phase-contrast microscopy (Olympus, Japan). As for the Alamar blue assay, the MDSC cells were seeded into a 96-well plate with GM at a density of 5000 cells per well. At each time point, culture medium was removed and cells were incubated with serum-free medium containing 10% (v/v) Alamar blue reagent for 4 h. Then, 100 μl of the medium of each well was transferred to a 96-well black plate, and the fluorescent intensity was measured in microplate reader (Thermo Fisher) at the wave length of 560/600 nm. Tests were repeated six times for each group, and the data was normalized to control group. For LIVE/DEAD staining, cells were cultured with LIVE/DEAD reagent for 1 h in the incubator according to the manufacturer’s instructions; images were obtained using a fluorescent microscope (Olympus, Japan). ELISA was performed as previously described [38]. Briefly, PGE2 ELISA kit (Abcam Inc., Cambridge, MA, USA) was used according to the manufacturer’s protocols. As a reference for quantification, standard curves were established using known concentrations of recombinant PGE2 proteins.

### Quantitative real-time polymerase chain reaction

All the procedures were conducted as previously reported [34]. Total RNA was extracted using the RNeasy Mini Kit (Qiagen, Valencia, CA, USA), and cDNA was reversely synthesized using the PrimeScript™ RT reagent kit (Perfect Real Time, TaKaRa, Japan). According to the manufacturer’s instruction, quantitative real-time polymerase chain reaction (qPCR) was performed in a 20-μl solution system containing 10 μl reaction mixture, 2 μl cDNA, and 300 nM gene-specific primers (myoD: forward: GGAGACATCCTCAAGCGATGC, reverse: GCACCTGGTAAATCGGATTG; myoG: forward: AACCCAGGAGATCATTTGC, reverse: GGAAGGTGACAGACATATCC; Myf5: foward: GGAATGCAATCCGCTACATT, reverse: CAGGGCAGTAGATGCTGTCA) [39]. The qPCR was performed in 7500 Real-Time PCR Detection System (Applied Biosystems, Irvine, CA, USA). Each sample was tested in triplicate. The relative mRNA expression levels were normalized to GAPDH as the endogenous normalization control.

### Western blot analysis

Western blot analysis was performed as previously reported [40]. The cultured cells were lysed with RIPA lysis buffer (Beyotime Institute of Biotechnology, China) supplemented with 1 mM PMSF (Invitrogen), and then, the protein concentration was determined using a BCA protein assay kit (Thermo Fisher). Equal amounts of proteins were separated by 10% SDS-PAGE electrophoresis and transferred to a 0.22-μm PVDF membrane (Millipore Corporation, Billerica, MA, USA). The membrane was blocked with 5% BSA for 1 h in room temperature and was then incubated with the following primary antibodies: anti-myoG (1:1000, Abcam), anti-myoD (1:1000, Abcam), anti-phospho Akt (1:1000, Abcam), anti-Akt (1:1000, Abcam), and anti-β-actin (1:3000, Abcam) at 4 °C overnight. Then, the membrane was incubated with an anti-rabbit (1:5000) or anti-mouse (1:5000) fluorescein-conjugated secondary antibody (Abcam). The immune bands were visualized by Odyssey V3.0 image scanning. All of the procedures were performed three times, and the bands were selected from individual results. Quantitative analysis was performed using ImageJ software; the normalized greyscale was calculated by dividing the individual greyscale of each marker to the β-actin.

### Immunocytochemistry

Immunocytochemistry cellular immunofluorescence was conducted as previously described [41]. The MDSCs were first fixed in 4% PFA (Sigma) and permeabilized with 1% Triton X-100 (Invitrogen). The cells were incubated with an optimal concentration of anti-MHC antibody, anti-CD146 antibody, anti-PAX7 antibody, and anti-PW1 antibody (1:200, all from Abcam) overnight at 4 °C, incubated with an Alexa Fluor 488 or 546 secondary antibody (1:800, Invitrogen), followed by rinsing three times with PBS. Then, cell nuclei were stained with Dapi (Invitrogen) prior to being imaged by fluorescent microscope (Olympus). The number, length, diameter, and maturation index data of myotubes were obtained from at least three images of each sample using ImageJ software, and cell positive rate of each marker was calculated by normalizing the positive cell number to the total cell number.

### Surgical procedure and gel casting

All animal surgery procedures were approved by the Institutional Animal Care and Use Committee of Nanjing Medical University. The procedure was performed on 8-week-old female SD rats (weight 150–180 g) according to a previously described rat model of tibialis anterior muscle defect under aseptic condition [35]. Briefly, the SD rats were anesthetized by intra-peritoneal injection of pentobarbital (Nembutal, 4 mg/100 g). To expose the tibialis anterior muscle, a longitudinal incision was made, followed by the creation of a wedge-shaped defect of 6 mm × 3 mm × 4 mm in length, width, and depth. For in situ casting of the MDSCs with fibrin gel, freshly harvested MDSCs (1 × 10^6^) were re-suspended in 20 μl of fibrinogen solution (MedChemExpress) with or without 100 nM of SW033291, and the muscle defect was filled with the solution followed by addition of thrombin solution (5 IU/2 μl, MedChemExpress) to induce gel casting [42]. After 2 min waiting period to ensure gel solidification, the wound was carefully covered with the fascia and skin and secured using sutures. A total of 9 rats with 18 muscle defects were randomly allocated into 3 groups: (1) gel group, the defect was filled with gel alone (*n* = 6); (2) gel/MDSC group, the defect was filled with gel incorporation with MDSCs (*n* = 6); and (3) gel/MDSC+SW033291 group, the defect was filled with gel containing 100 nM SW033291 and MDSCs. Another 3 rats with 6 healthy limbs were used for mechanical measurements as control limbs.

### Mechanical measurement

Following 4 weeks of recovery, muscle contractile properties were measured in accordance with the literature [43, 44]. Briefly, animals were anesthetized with an intra-peritoneal injection of pentobarbital to maintain an adequate anesthesia throughout the procedures. The animal was placed on a temperature-controlled platform to maintain body temperature, and the hindlimb was fixed. The whole tibialis anterior muscle was carefully isolated without damaging the nerve and blood supply; a suture was tied around the distal tendon before the tendon was severed and tied to the servo motor (Aurora Scientific). The muscle was activated by stimulation of the sciatic nerve using a bipolar platinum wire electrode. The voltage of single 0.2 ms stimulation pulses was adjusted to give a maximal isometric twitch, and the muscle length was adjusted to the optimal length at which twitch force was maximal. With the muscle at optimal length, 300 ms stimulus pulses were used with increasing stimulation frequencies until the maximum isometric tetanic force was achieved. After that, whole tibialis anterior muscle was removed and muscle mass was measured; specific muscle force was calculated by normalizing maximal force to muscle mass.

### Histomorphological analysis and immunohistochemistry

The histological sections with hematoxylin-eosin (HE), Masson, and immunohistochemistry staining were employed to analyze the regeneration of skeletal muscle tissue according to reported literature [45, 46]. The tibialis anterior muscles with the defect site were harvested and fixed in 4% formalin then embedded in paraffin. The cross-section of the central area of each sample was sliced using a microtome (Leica, Hamburg, Germany) and stained following the procedure of hematoxylin-eosin staining kit (Invitrogen) and Masson staining kit (Abcam). The regenerated tissues and fibrosis were observed using an optical microscope (Olympus). Immunohistochemistry was performed using the primary anti-mouse or anti-rabbit antibodies against GFP, vWF, and CD68 (1:100, all purchased from Abcam), followed by goat anti-mouse IgG Alexa Fluor 596 or goat anti-rabbit IgG Alexa Fluor 647 (1:400, Abcam). Finally, the sections were stained with Hoechst (1:1000, Abcam) prior to be imaged by fluorescent microscope (Olympus). The quantitative results were analyzed using ImageJ software with three randomly selected fields of each section and three sections per specimen.

### Statistical analysis

Each experiment was performed at least three times, and the data were presented as mean ± standard deviation. Statistical significance was determined by unpaired Student’s *t* test, and a value of *P < 0.05 was considered to be statistically significant.

## Results

### Characterization of rat MDSCs

**Fig. 1:**
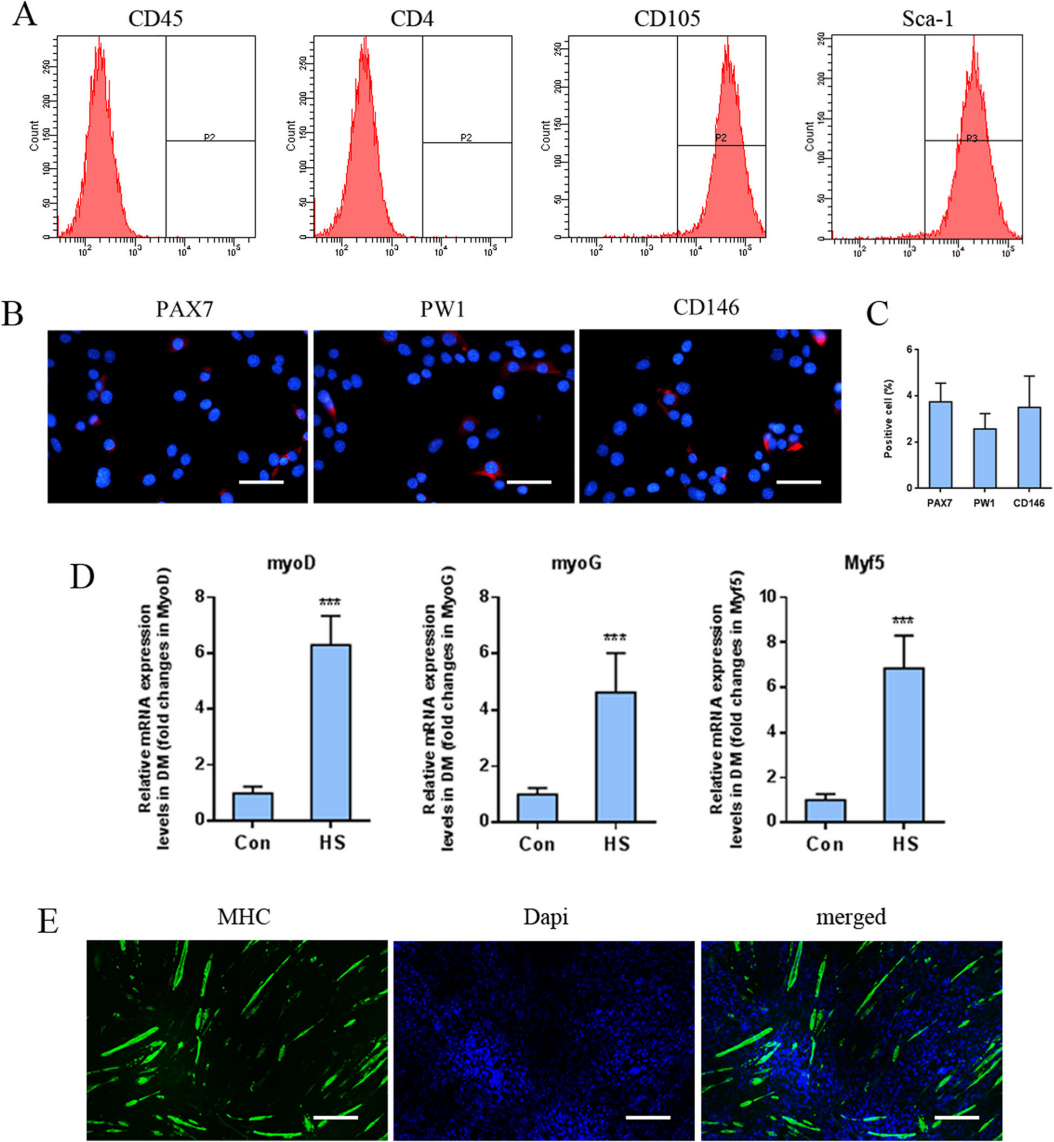
**a** Flow cytometry of the cellular surface markers of CD45, CD4, CD105, and Sca-1 in MDSCs. **b** Cellular immunochemistry analysis of satellite cell marker PAX7, pericyte marker PW1, and interstitial progenitor cell marker CD146 expression in MDSCs, scale bar: 50 μm. **c** Positive cell rate calculation based on fluorescent imaging. **d** qPCR analysis of the myogenic marker expression in MDSCs under myogenic induction on day 3. **e** Cellular immunochemistry image of MHC expression (green) in MDSCs and nuclei stained with Dapi (blue), scale bar 200 μm. ***P < 0.001

To characterize rat muscle-derived stem cells (MDSCs), flow cytometry was used to detect the expression levels of cellular surface markers. Rat MDSCs showed high expression of CD105 (95.7 ± 2.43%) and Sca-1 (92.3 ± 3.48%), whereas CD4 (1.8 ± 0.63%) and CD45 (2.3 ± 1.1%) were rarely detected (Fig. 1a). Immunostaining was also utilized to distinguish MDSCs from other muscle-derived progenitor cells (Fig. 1b, c). The majority of MDSCs did not express PAX7 (key marker for satellite cells, 3.75 ± 0.81%), PW1 (key marker for pericytes, 2.57 ± 0.66%), and CD146 (key marker for interstitial progenitor cells, 3.50 ± 1.36%). Furthermore, to investigate the myogenic differentiation potential, MDSCs were cultured in differentiation medium containing 2% horse serum; then, quantitative real-time polymerase chain reaction (qPCR) was performed to detect myogenic-specific gene expression, along with immunocytochemistry for the detection of myosin heavy chain (MHC) expression. qPCR results showed that the expression of myogenesis-related genes such as myoD, myoG, and Myf5 increased under myogenic induction as compared to the control (Fig. 1d). Similarly, the elevated expression of MHC was also observed in MDSCs along with the morphological changes of MDSCs toward myotube (Fig. 1e). Taken together, these data demonstrated that rat MDSCs possessed specific phenotype characteristics and myogenic differentiation potential.

### Biological activity and cytotoxicity evaluation

**Fig. 2:**
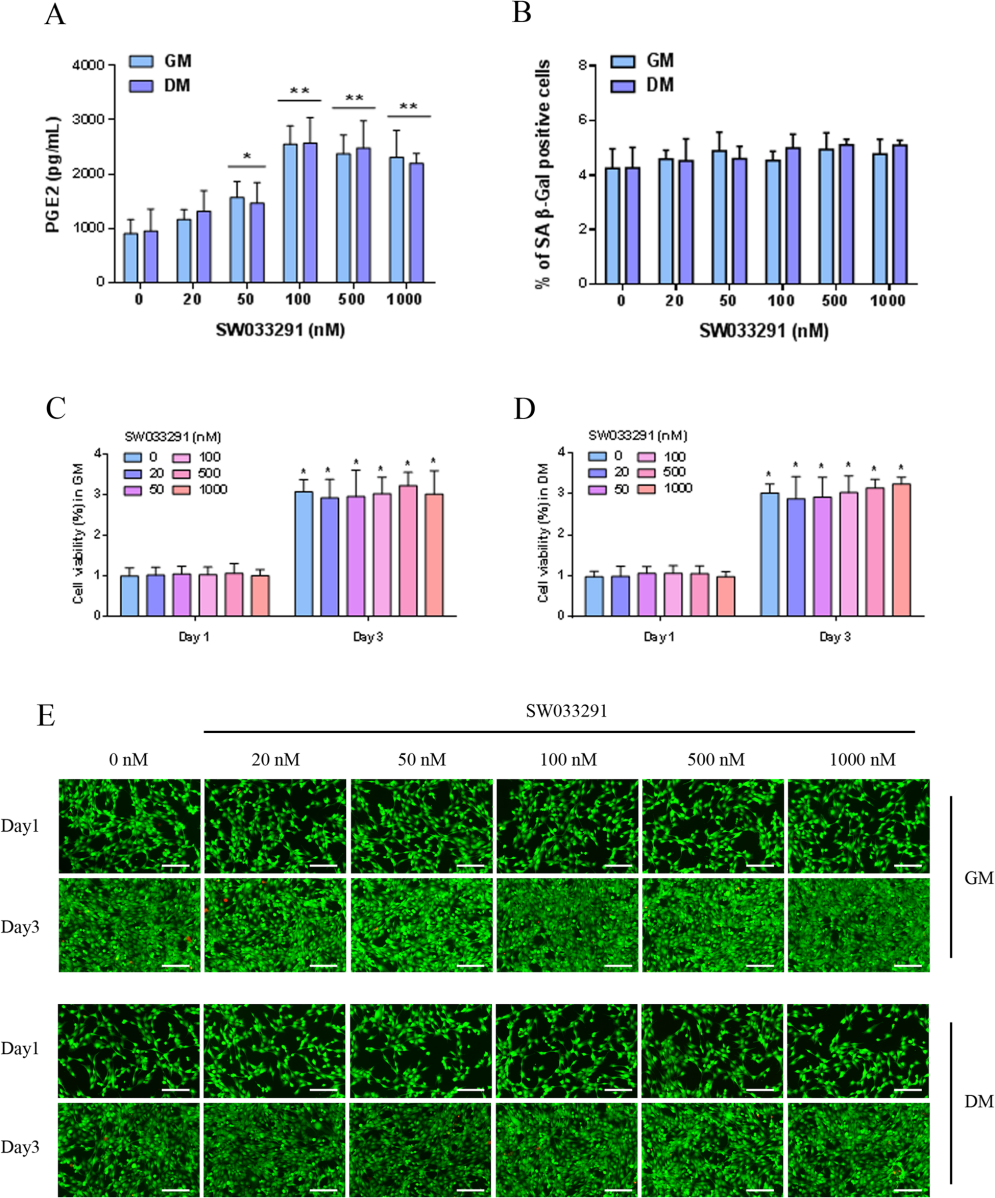
**a** PGE2 level produced by MDSCs cultured in DM and GM treated with different concentration of SW033291. **b** Percentage of SA-β-Gal positive MDSCs in DM and GM treated with different concentration of SW033291. **c** Cell viability of MDSCs in GM and **d** DM under different concentration of SW033291 treatment on day 1 and day 3. **e** LIVE/DEAD staining of MDSCs cultured in DM and GM treated with different concentration of SW033291 on day 1 and day 3; live MDSCs stained in green and dead cell in red, scale bar 100 μm. *P < 0.05, **P < 0.01

To investigate the biological effects of SW033291 on prostaglandin E2 (PGE2) production, MDSCs were cultured with an ascending concentration of SW033291 from 20 to 1000 nM in both growth medium (GM) and differentiation medium (DM). PGE2 contents in GM and DM were firstly tested, and the result showed that PGE2 was under the detectable level. After MDSCs were incubating in the medium for 24 h, ELISA was used to detect the level of PGE2 in the conditioned medium. The baseline production of PGE2 by MDSCs had no significant difference between GM and DM with a detected concentration of 905.08 ± 225.92 and 952.75 ± 400.15 pg/ml, respectively. The addition of SW033291 increased PGE2 production in a dose-dependent manner without significant difference between DM and GM; significant elevation of approximately 2.8-fold was observed in 100 nM group, reaching 2548.23 ± 341.16 and 2569.22 ± 471.24 pg/ml, followed by 500 nM and 1000 nM group (Fig. 2a). To test the effects of SW033291 on cell senescence, senescence-associated β-galactosidase (SA β-Gal) staining was performed; our results showed that MDSCs treated with different concentration of SW033291 displayed minimal SA β-Gal staining with a cell positive rate around 5%, and there was no significant difference among each treatment group and between GM and DM (Fig. 2b). We further evaluated the cytotoxicity of SW033291 on MDSCs in both GM and DM. As shown in cell viability assay (Fig. 2c, d), no obvious negative effect of SW033291 on MDSC viability was observed with the ascending concentration from 20 to 1000 nM, and the difference was statistically insignificant among various groups and between different culture medium. After 72 h, cell viability in different group significantly increased, which indicated favorable cell proliferation capacity; no significant difference was observed among each group and between different culture medium. The LIVE/DEAD staining was also performed to show that most MDSCs were live (stained as green) in each group after incubating for 24 h; only very few dead cells (stained as red) were found. After 72 h, the number of green MDSCs significantly increased in various groups, showing favorable proliferation capacity, and no significant difference was observed among various groups and between DM and GM (Fig. 2e). Taken together, our data showed that SW033291 increased the production of PGE2 by MDSCs both in GM and DM with the best response reached approximately 2.8-folds increase, and MDSCs were well-tolerated to SW033291 in various concentrations with strong proliferation potential.

### In vitro myogenic differentiation analysis

**Fig. 3:**
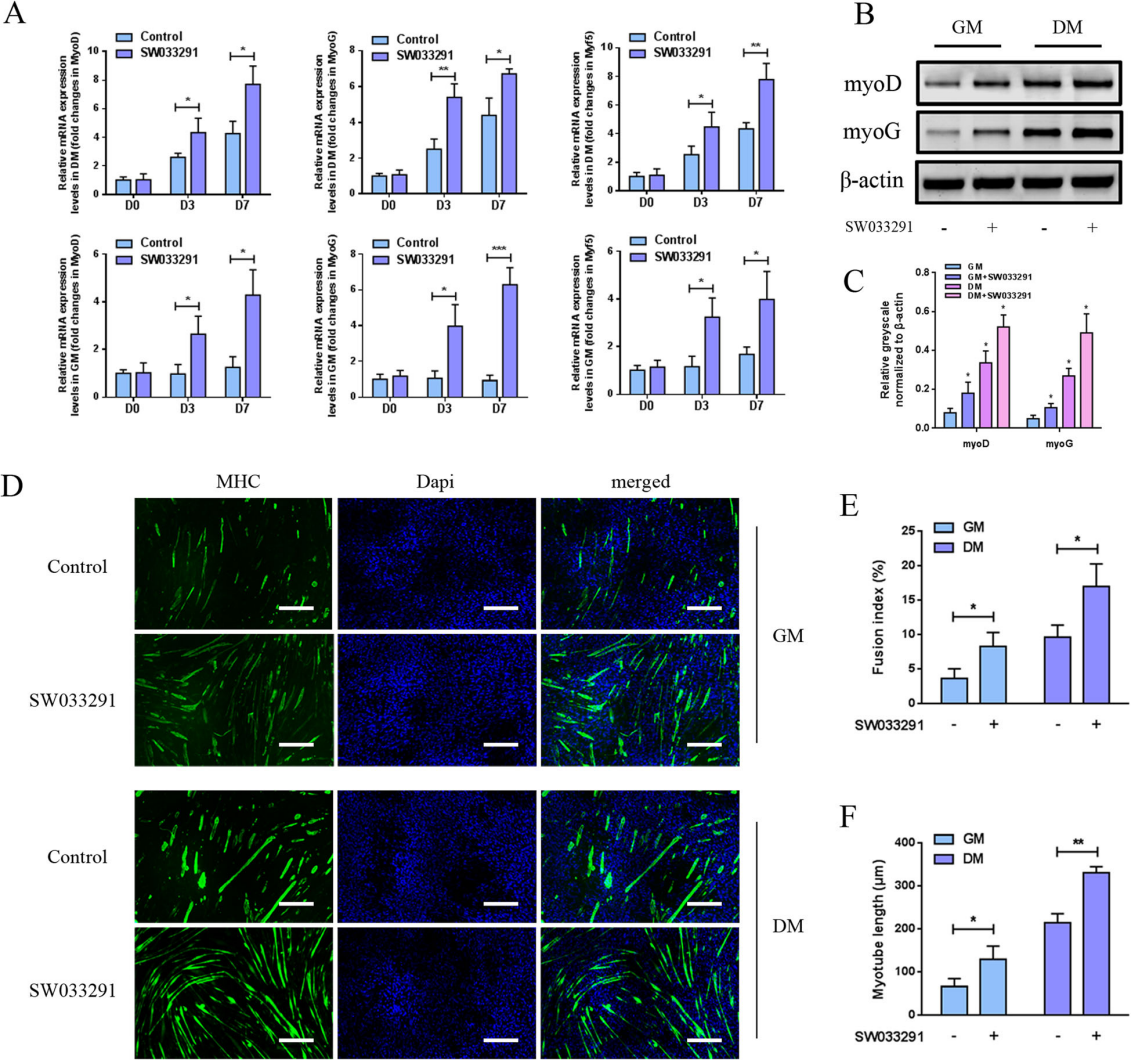
**a** qPCR analysis of the expression of myogenic differentiation-related genes in MDSCs cultured in DM (top row) and GM (bottom row) under 100 nM SW033291 treatment on days 3 and 7. **b** Western blot analysis of the protein expression of myogenic markers in MDSCs cultured in DM and GM under 100 nM SW033291 treatment on day 3. **c** Quantitative analysis of western blot bands. **d** Cellular immunochemistry image of MHC in MDSCs cultured in DM and GM under 100 nM SW033291 treatment on day 7, scale bar 200 μm. **e** Fusion index and **f** myotube length were calculated based on fluorescent images. *P < 0.05, **P < 0.01

To address whether SW033291 could regulate MDSC myogenic differentiation, MDSCs were cultured in either GM or DM in the presence of SW033291 at a final concentration of 100 nM; then, qPCR and western blot were performed on days 0, 3, and 7. The mRNA expression levels of myogenic factors as myoG, myoD, and Myf5 in MDSCs cultured in DM showed a time-dependent increase, of note is that the addition of SW033291 drastically boosted the expression of these markers as compared to the control group at each time point. As for MDSCs cultured in GM, SW033291 also significantly enhanced myogenic gene expression on days 3 and 7 as compared to the control group (Fig. 3a). Western blot analyses on day 3 further revealed that the protein levels of myoG and myoD in MDSCs cultured in both DM and GM were elevated by SW033291 as compared to the control (Fig. 3b, c). To detect the MHC expression and myotube formation during SW033291 induction, immunocytochemistry staining of MHC protein in MDSCs in both DM and GM was performed. Figure 3d showed the immunofluorescent staining images of MHC in MDSCs after incubating with 100 nM SW033291. As Fig. 3 e and f showed, after 7 days incubation in DM, myotube formation was observed in both groups; however, as compared to the control, SW033291 significantly improved myotube formation as exhibited by higher fusion index (17.05 ± 3.26% vs 9.68 ± 1.74%, *P* < 0.05) and myotube length (331.81 ± 14.01 μm vs 215.33 ± 20.97 μm, *P* < 0.01). As for MDSCs cultured in GM, similar patterns were observed with the fusion index (8.32 ± 2.02% vs 3.65 ± 1.43%, *P* < 0.05) and myotube length (130.24 ± 30.75 μm vs 67.12 ± 18.07 μm, *P* < 0.05). Collectively, these findings indicated that the addition of 100 nM SW033291 significantly enhanced in vitro myogenic differentiation and myotube formation of MDSCs in both DM and GM.

### In vitro myogenesis mechanism investigation

**Fig. 4:**
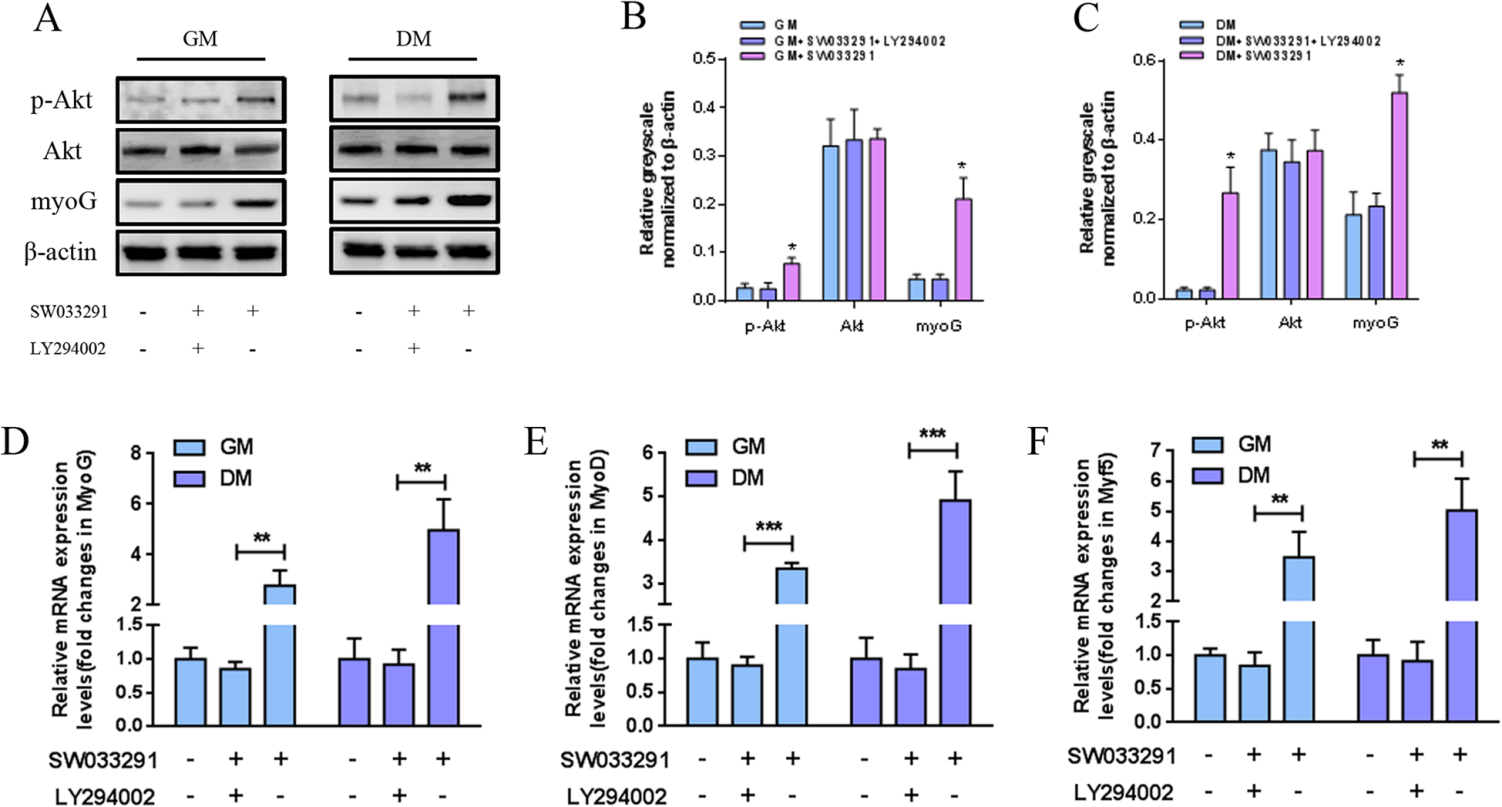
**a** Western blot analysis of the protein expression of myogenic markers and Akt in MDSCs under 100 nM SW033291 treatment with or without LY294002 on day 3. **b**, **c** Quantitative analysis of western blot bands. **d**–**f** qPCR analysis of the expression of myogenic differentiation-related genes in MDSCs under 100 nM SW033291 treatment with or without LY294002 on day 3. **P < 0.01, ***P < 0.001

**Fig. 5:**
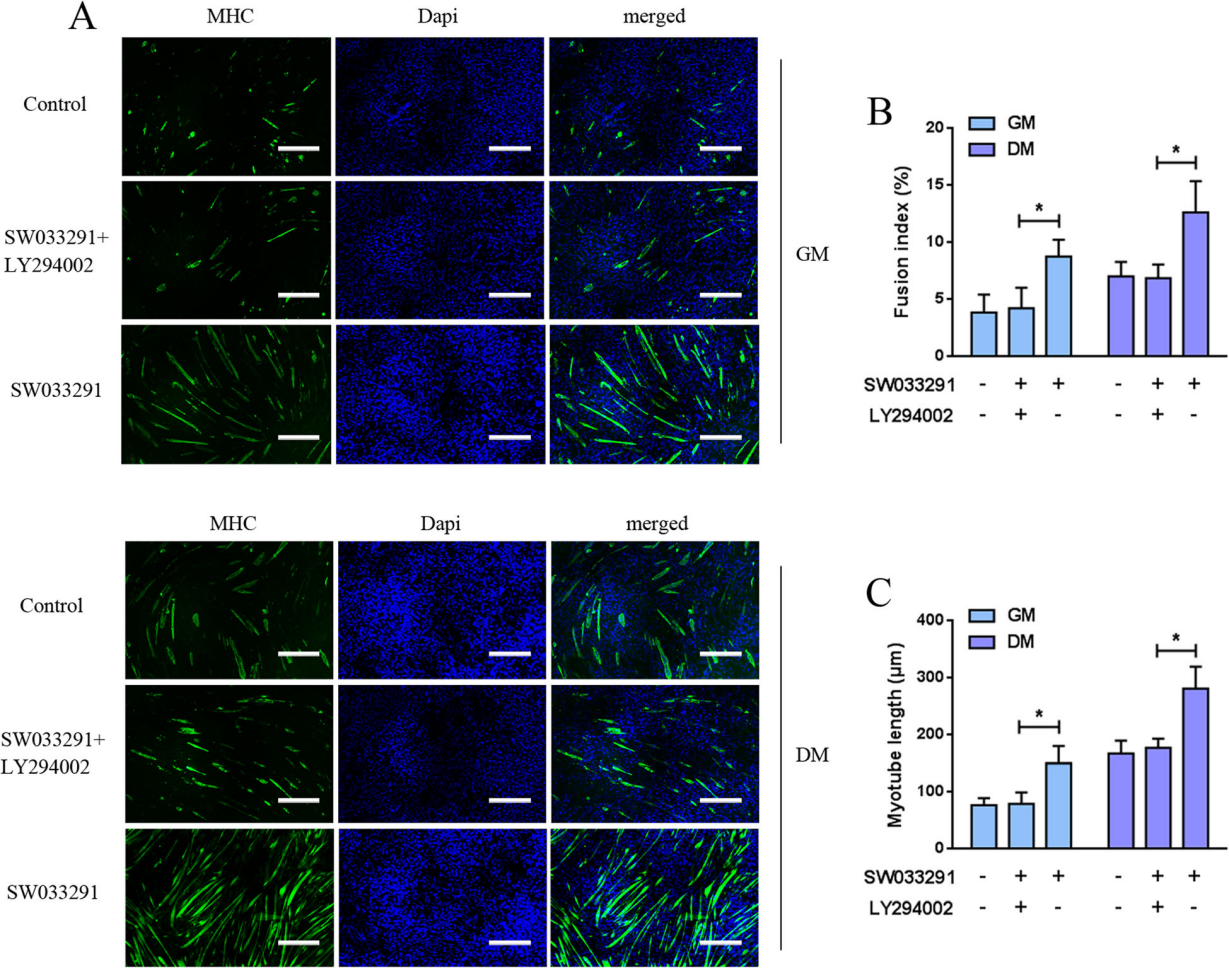
**a** Cellular immunochemistry images of MHC in MDSCs under 100 nM SW033291 treatment with or without LY294002 on day 7. **b** Fusion index and **c** myotube length were calculated based on fluorescent images, scale bar 200 μm. *P < 0.05

Our data showed that SW033291 enhanced the production of PGE2 by MDSCs and promoted the myogenic differentiation and myotube formation. As the PGE2/EP4 activation positively regulates PI3K/Akt pathway, and the pathway has been proved to be essential in myogenesis, thus, we wondered whether SW033291 mediated MDSC myogenesis through PI3K/Akt pathway. To test this hypothesis, immunocytochemistry, qPCR, and western blotting assay were performed in MDSCs cultured in GM or DM with or without the inhibition of PI3K/Akt pathway by specific inhibitor LY294002. Western blot analysis on day 3 showed that SW033291 activated PI3K/Akt pathway through promoting the phosphorylation of Akt as compared with the control. However, in the presence of PI3K/Akt pathway inhibitor LY294002, both phospho-Akt and myoD levels in MDSCs were suppressed as compared with SW033291-treated cells (Fig. 4a–c). This was further validated by qPCR on day 3; our data showed that the mRNA expression levels of myogenic markers as myoD, myoG, and Myf5 were decreased significantly in MDSCs treated with the combination of SW033291 and LY294002 when compared with SW033291 alone (Fig. 4d–f). As for MHC expression and myotube formation, in the absence of LY294002, after incubation with 100 nM SW033291 for 7 days, myotubes with high length and fusion index were observed both in DM and GM (Fig. 5a). However, after adding LY294002, the myotube formation was significantly decreased as indicated by lower fusion index and myotube length (Fig. 5b, c). Taken together, these results indicate that the SW033291 regulated in vitro myogenic differentiation and myotube formation of MDSCs through activating PI3K/Akt signaling pathway.

### In vivo muscle regeneration

**Fig. 6:**
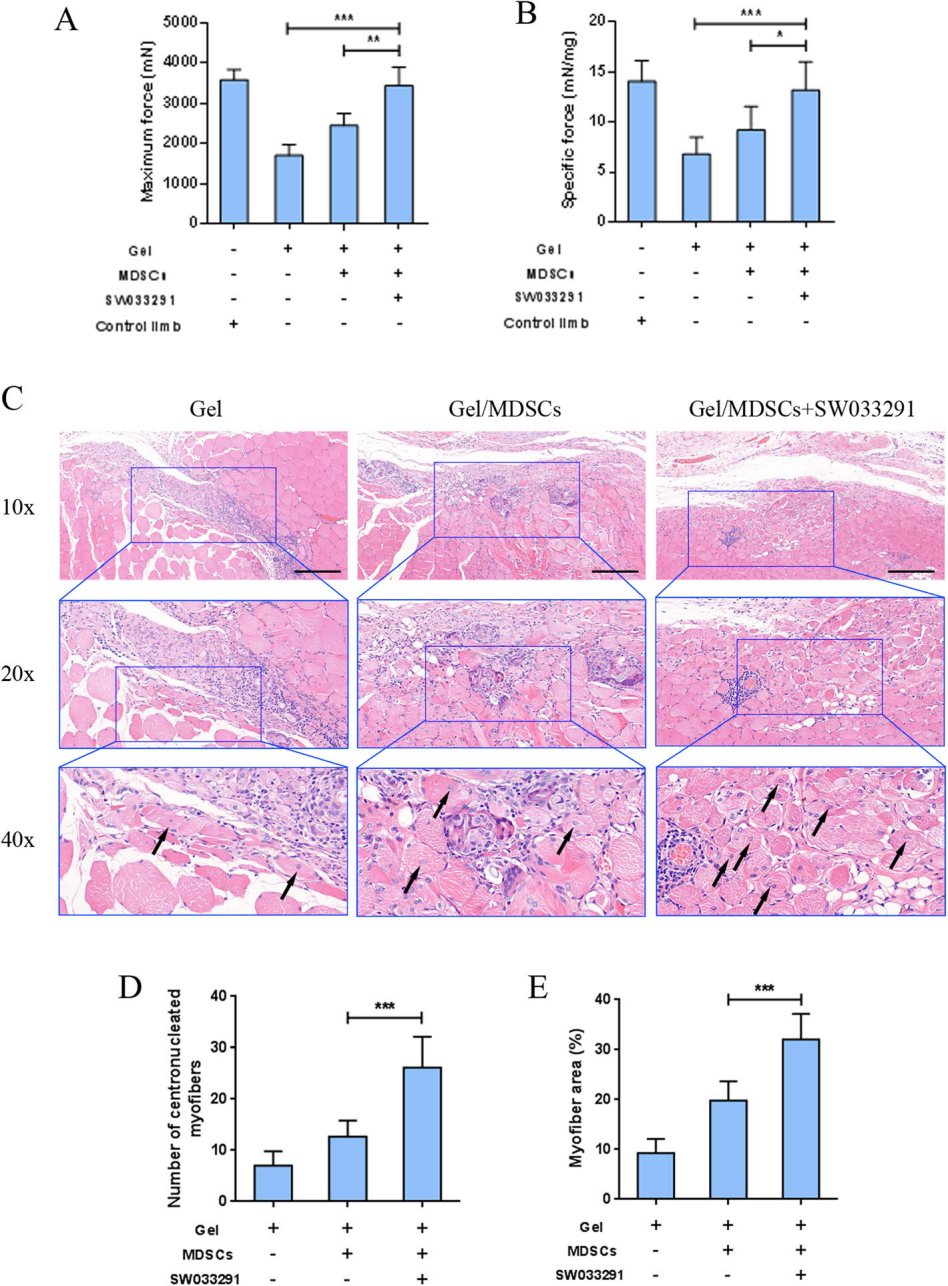
**a** Mechanical measurements to evaluate functional recovery of tibialis anterior muscle as reflected by maximum muscle force and **b** specific force by normalizing maximum force to the muscle mass. **c** Representative histological sections by hematoxylin-eosin stained. **d** Number of centronucleated myofibers and **e** percentage of newly formed muscle tissue were calculated based on histological sections, scale bar 100 μm. ***P < 0.001

To investigate the in vivo muscle regeneration capacity of SW033291, a rat tibialis anterior muscle defected model was created, and the compound was incorporated with MDSCs into fibrin gel to repair the defect by in situ casting. Eighteen muscle defects were randomly allocated into the three groups: (1) gel group, the defect was filled with gel alone; (2) gel/MDSC group, the defect was filled with gel incorporation with MDSCs; and (3) gel/MDSC+SW033291 group, the defect was filled with gel containing 100 nM SW033291 and MDSCs. Four weeks after surgical procedures, the regenerated muscle was evaluated by mechanical measurement and histological examinations. The maximal force produced by the gel group (1700.48 ± 276.39 mN) was significantly lower than those produced by the gel/MDSC group (2448.40 ± 303.79 mN) and gel/MDSC+SW033291 group (3441.42 ± 452.38 mN) (*P* < 0.001), and no significant difference was observed between the gel/MDSC+SW033291 group and the control limb (3572.26 ± 256.97 mN) (Fig. 6a). The specific forces calculated by normalizing maximal force to muscle mass showed similar trends (Fig. 6b). These results indicated that fibrin gel with or without MDSCs did not fully restore maximum force production to the injured tibialis anterior muscles, and the incorporation of SW033291 dramatically improved functional muscle regeneration to the level as comparable to non-injury muscle. Regenerated muscle tissues were observed in all groups as shown by newly formed myofibers with the nucleus located at the center (Fig. 6c); however, when compared to the gel group (7.00 ± 2.83) and gel/MDSC group (12.67 ± 3.14), significantly increased number of centronucleated myofibers appeared in the HE-stained sections of the gel/MDSC+SW033291 group (26.17 ± 5.95) (*P* < 0.001) (Fig. 6d). Furthermore, histomorphological analysis showed significantly larger area of regenerated myofibers in the gel/MDSC+SW033291 group (32.02 ± 5.16%) as compared with the gel+MDSC group (19.88 ± 3.57%) and gel group (9.24 ± 2.88%) (*P* < 0.001) (Fig. 6e).

**Fig. 7:**
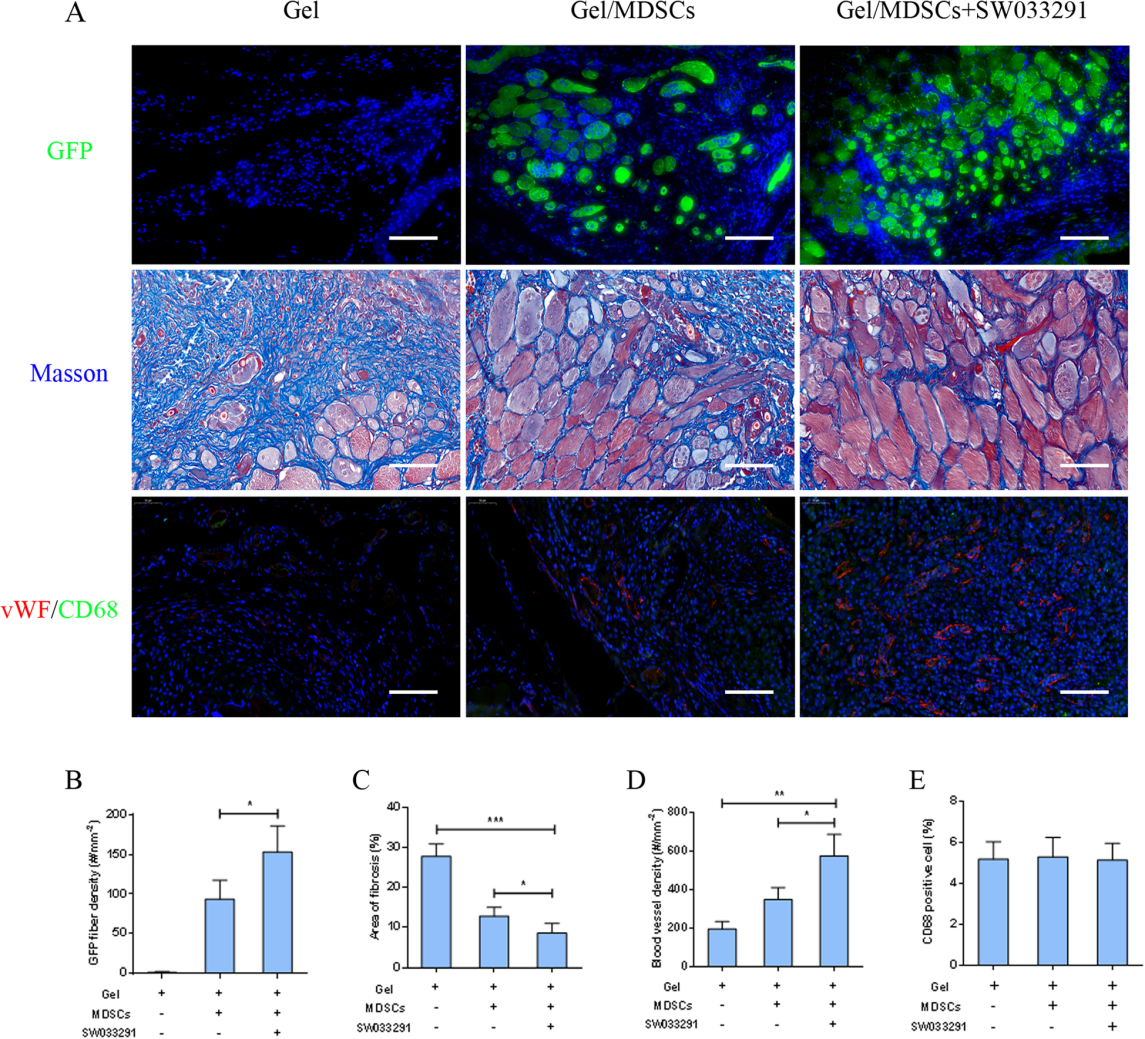
**a** Immunohistochemistry analysis of GFP-positive myofibers within the defect region (first row). Masson staining of fibrotic collagen deposition in the defect region (second row). Immunohistochemistry analysis of vascularization using vWF-stained blood vessel within the defect region (third row). Immunohistochemistry analysis of immune response using CD68 positive cells within the defect region (third row), scale bar 50 μm. **b**–**e** Quantitative analysis based on imaging. *P < 0.05, **P < 0.01, ***P < 0.001

To monitor the fate of implanted MDSCs in the defect sites, immunohistochemical analysis of GFP-positive fiber density was performed as shown in the first row of Fig. 7a. GFP was positively stained in the regenerated myofibers within the defect region of the gel/MDSC group (94 ± 24/mm^2^) and gel/MDSC+SW033291 group (153 ± 32/mm^2^); however, no GFP-positive staining was observed in the gel group (Fig. 7b). These data suggested that the implanted MDSCs contributed to the regeneration of muscle in the defect region. Masson staining was used to display fibrotic collagen deposition in the extracellular matrix shown in the second row of Fig. 7a. The fibrotic staining area in the gel/MDSC+SW033291 group (8.66 ± 2.39%) was significantly lower than the gel/MDSC group (12.88 ± 2.22%) (*P* < 0.05) and gel group (27.93 ± 3.10%) (*P* < 0.001) (Fig. 7c). Moreover, the regenerated blood vessels were also confirmed by immunohistochemical staining using vWF as a specific marker of endothelial cells to identify blood vessels (third row of Fig. 7a). The density of vWF-labeled blood vessel in different groups showed similar tendency as in descending order of 576 ± 112/mm^2^ in the gel/MDSC+SW033291 group, 349 ± 62/mm^2^ in the gel/MDSC group, and 196 ± 39/mm^2^ in the gel group (*P* < 0.05) (Fig. 7d). As for the immune response during the regeneration procedure, CD68 was used as a marker to detect M2 macrophage (third row of Fig. 7a); our results showed that macrophage infiltration was mild in all three groups, and no significant differences were observed among them (*P* > 0.05) (Fig. 7e). Taken together, our data indicated that fibrin gel incorporated with MDSCs resulted in muscle regeneration with mild immune response; more importantly, the adding of SW033291 significantly promoted functional muscle regeneration with less fibrosis and sufficient vascularization, suggesting that SW033291 played as a positive regulator in the process of skeletal muscle repair in vivo.

## Discussion

A large number of essential regulatory factors have been identified for affecting the regeneration of skeletal muscle, and the complex underlying mechanisms involved motivate researchers to further explore. Prostaglandin E2 (PGE2), a potent inflammatory cytokine produced by multiple cell types and released to the stem cell niche after muscle injury, has recently emerged as key regulator which promotes myogenic stem cell expansion and muscle regeneration, showing great potential as therapeutic target in repairing large skeletal muscle defect [47]. Among those efforts that have been made toward the elevation of PGE2, SW033291 stands out as a promising candidate. It is a small-molecule inhibitor targeting the PGE2 degrading enzyme 15-hydroxyprostaglandin dehydrogenase (15-PGDH), which potently elevate the secretion of PGE2 both in vitro and in vivo [29]. We wondered whether SW033291 treatment could increase PGE2 production of muscle-derived stem cells (MDSCs). The biological activity of SW033291 in promoting PGE2 production was firstly reported using different concentrations including 25, 50, 100, 250, 500, and 1000 nM, and a dose-dependent increase of PGE2 was observed with the EC_50_ (concentration for 50% of maximal effect) between 50 and 100 nM and the maximum effect at 500 nM (approximately 3-fold increase) [29]. Antczak and colleagues further investigated compound-induced PGE2 production using 4, 20, 100, 500, and 2500 nM. According to their data, SW033291 could lead to PGE2 increase from a low dose of 20 nM (1.9-fold increase) to a saturation dose of 2500 nM (2.7-fold increase). Of special notice is that the extent of PGE2 increase after 100 nM in the previous study was relatively low, suggesting that the biological effects might approach saturation [48]. In our study, we defined the dose selection range based on previously reported data and treated MDSCs with SW033291 from 20 to 1000 nM to explore the biological effects of SW033291. Our results indicated that the production of PGE2 showed a dose-dependent manner; a maximal effect was observed in the 100 nM group with a 2.8-fold increase compared to the non-treated MDSCs. These data demonstrated that treatment of SW033291 on MDSCs could significantly promote PGE2 production.

It has been well-established that extracellular stresses, i.e., chemical, physical, and biological factors, might have impact on various cellular behaviors including cell proliferation, differentiation, apoptosis, and senescence. In particular, it has been demonstrated that stem cells are prone to senescence rather than apoptosis even after high exogenous stress [49]. Our data indicated that SW033291 promoted PGE2 production, and accumulating evidence suggests that PGE2 plays a key role in inflammatory processes and is an important component of the aging process, which could induce cellular senescence in fibroblast [50–52]. To investigate the effects of SW033291 on MDSC senescence, we calculated senescence-associated β-galactosidase (SA β-Gal)-positive cells. Our data revealed that the percentage of SA β-Gal-stained MDSCs maintained a relatively low level among each group, suggesting that SW033291 had very limited effects on cell senescence. This might be attributed to the fact that SW033291 only induced the maximum PGE2 production to a concentration of 2.5 ng/mL (equals to ~ 8 nM) in the medium, which is significantly lower than previously reported extracellular level to induce SA β-Gal marker [50, 52], and literature also shows that 1–10 nM PGE2 only results in 5% SA β-Gal-positive cells [51]. These data suggested that SW033291-induced PGE2 production level was within physiological range, and it had very limited effects on cell senescence. Besides, previous in vivo systemic injection of SW033291 in mice showed well-tolerated safety profile [29], and our cytotoxicity results also demonstrated that no obvious cytotoxic effects appeared in all concentration groups and MDSCs treated with SW033291 possessed favorable proliferation potential. Collectively, these results illustrated that MDSCs were well-tolerated to the small-molecule inhibitor SW033291, which could serve as a potent inducer for the production of PGE2 by MDSCs.

The advantage of employing MDSCs for muscle regeneration in our study is based on its large quantity, relative easy accessibility, strong proliferation, and myogenic differentiation potential. Although satellite cells (SCs) have been widely studied owing to their pivotal role in endogenous muscle repair, regeneration, and commitment to the myogenic lineage, SCs make up only a small proportion of the muscle cell population [53]. As one of the major concerns for stem cell-based therapy, it is the very limited source that restricts the wide application in muscle regeneration. As a population of progenitors distinct from SCs, MDSCs have been identified to reside in the interstitial spaces of muscle tissue. According to the histological finding, they appear to be associated closely within the vascular and perivascular regions in the muscle, although some of the MDSCs were located beneath the basal lamina of myofibers where SCs normally reside, the majority of MDSCs were located in the areas that are occupied by capillaries [54–57]. MDSCs are a heterogeneous mixture of cells with variable proliferation and differentiation potentials; currently, no standard marker has been set for MDSCs, and more work thus needs to be performed to establish differences and similarities between the various populations. The more recent characterization of MDSCs by various groups has identified a panel of cellular markers to distinguish MDSCs from other muscle originated progenitor cells including SCs, pericytes, and interstitial progenitor cells [33, 58, 59]. Our data aligned with literature that the isolated MDSCs showed high expression in CD105 and Sca-1, whereas low expression in CD4 and CD45; it also showed negative staining for the SC-specific marker PAX7, the pericyte-specific marker CD146, and interstitial progenitor cell marker PW1. Although better understanding about the nature of MDSCs is required, they have been acceptable for cell-based therapeutic applications with the unique advantage to overcome cell source limitation to some degree. They can be isolated from muscle tissues and exponentially expanded in vitro; more importantly, they can maintain strong proliferation and differentiation potential [33]. Our in vitro results clearly demonstrated that the myogenic induction significantly promoted myogenic-specific gene expression and the myotube fusion of MDSCs. LIVE/DEAD staining further indicated that MDSCs possessed strong proliferation potential in both differentiation medium (DM) and growth medium (GM). These data supported that MDSCs could be served as promising seed cells in repair of muscle defects.

Various studies have indicated that PGE2 plays pivotal role in regulating muscle regeneration. PGE2 is synthesized from arachidonic acid by cyclooxygenase-2 (COX-2); the ablation of COX-2 in myogenic precursor cells leads to decreased myotube formation, and this can be rescued by adding PGE2 into the culture medium [60]. Another research revealed that PGE2 augments muscle-specific stem cell (MuSC) proliferation through PGE2/EP4 interaction and subsequently results in enhanced regeneration [27, 28]. These evidences motivated us to investigate whether the SW033291 treatment could contribute to the regulation of MDSC-mediated muscle regeneration and to explore the molecular mechanism. Based on our results about the biological effects and cytotoxicity of SW033291, 100 nM was selected for the following experiments due to its maximal induction effect and safety performance. Our data demonstrated that both mRNA and protein levels of myogenic markers in MDSCs were significantly increased by the treatment of SW033291, and myotube fusion showed similar patterns as augmented by SW033291. These results indicated that SW033291 played a positive role in regulating MDSC myogenic differentiation and myotube formation. As indicated by previous reported researches, the myogenic differentiation was driven by the activation of PI3K/Akt signaling pathway [61], and the upstream PGE2/EP4 interaction manipulates PI3K/Akt pathway activity [62]. Based on these evidences, we speculated that the promotive effects of SW033291 might exhibit through affecting PI3K/Akt pathway. Our western blot results showed that SW033291 could promote MDSC myogenic differentiation and simultaneously activated PI3K/Akt pathway by promoting the phosphorylation of Akt. However, the enhancing effects of SW033291 on myogenic differentiation and myotube formation were significantly attenuated by the adding of PI3K/Akt-specific inhibitor LY294002, suggesting that SW033291 might exerted its myogenic promotive effects through modulating PI3K/Akt pathway. These novel findings provided new insights into the regulatory role of SW033291 in potentiating MDSC myogenic differentiation, further demonstrating that the manipulation of PGE2 signaling not only affected the proliferation of myogenic precursor cells but also had impacts on their differentiation.

The delivery of seed cells by biomaterial scaffold is a highly promising strategy because the synergistic therapeutic potential for functional muscle regeneration has been proved in previous researches [63]. The incorporation of precursor cells with myogenic differentiation potential into supportive scaffolds has resulted in increased muscle regeneration at the site of muscle defect. Among the various biomaterials, fibrin gel has been one of the most studied materials due to its favorable biocompatibility with natural degradable property, and fibrin has its own advantage in supporting wound healing as indicated by researches demonstrating its role in promoting cell expansion, migration, and differentiation [64]. However, fibrin gel alone fails to significantly improve large muscle defect repair; thus, a novel approach of direct in situ casting of fibrin gel with MDSCs has been developed, which provides a feasible way for highly efficient and straightforward delivery of the scaffold/seed cell composite to the defect region [42]. In our study, due to the excellent physicochemical properties of small-molecule inhibitor SW033291 and fibrin gel, sufficient amount of MDSCs could be incorporated into fibrin gels with 100 nM SW033291 to form a uniform composite, which fully occupied and adhered to the defect. Mechanical measurement indicated that the incorporation of SW033291 into gel/MDSCs complex exhibited a significantly greater return of function than the other experimental conditions as reflected by enhanced maximal and specific force generated by the anterior tibialis. Histological examination analysis showed larger number of centronucleated myofibers and larger area of regenerated muscle tissues, together with mild immune response, less fibrosis, and sufficient vascularization. These observations should partly contribute to the sufficient MDSCs delivered to the defect region by in situ gel casting, because the lack of seed cells often resulted in impaired regeneration with severe scar formation; just as we observed, the implanted MDSCs contributed to the regeneration of muscle tissue and gel/MDSCs group showed much higher regenerative capacity and less fibrosis when compared to the gel group. More importantly, the loading SW033291 that greatly improved MDSC myogenic differentiation and myofiber formation within the defect region might play essential role in promoting muscle defect repair in this case. On the other hand, the better vascularization could partially support muscle regeneration because PGE2 has been associated in promoting angiogenesis in the wound healing microenvironment [65]. As for the immune response, CD68 was used to detect M2 macrophage because the first wave of M1 infiltration normally happens at early stage (peak at 3 days); then, macrophages undergo a phenotype switch toward M2 with functional alteration to support tissue repair [66]. In our study, the macrophage infiltration was mild, and no significant difference was observed among different groups; this observation might owe to the good compatibility of the exogenous implant complex including fibrin gel, MDSCs, and small molecule inhibitor, which only led to limited immune response within the defect region, and this further supported its application in muscle regeneration. Considering the above results, this in situ fibrin gel casting strategy with MDSCs and SW033291 incorporated exhibited promise for the repair of muscle defect.

## Conclusion

In summary, the results of this study showed that (1) the production of PGE2 by MDSCs were increased by a cohort of SW033291 with an ascending concentration from 20 to 1000 nM, among which 100 nM SW033291 showed the best effect along with little cytotoxicity. (2) The treatment of SW033291 significantly promoted myogenic differentiation and myotube formation of MDSCs both in DM and GM; these promotive effects of SW033291 were attenuated by the adding of PI3K/Akt pathway inhibitor LY294002. (3) The incorporation of SW033291 and MDSCs into fibrin gel showed enhanced functional muscle regeneration potential as demonstrated by higher number of centronucleated myofibers and area of regenerated muscle tissues, along with sufficient blood supply and less fibrosis. These data suggested that SW033291 enriched fibrin gel and MDSCs composite represented an effective and promising strategy for repairing muscle defect and provided preclinical data supporting its potential application in curing muscle-related diseases.

## Data Availability

All data generated or analyzed for this study are included in this published article.

## Abbreviations

15-PDGH: 15-Hydroxyprostaglandin dehydrogenase
COX: Cyclooxygenase
DM: Differentiation medium
DMSO: Dimethyl sulfoxide
EC_50_: Concentration for 50% of maximal effect
EP4: E-type prostanoid receptor 4
GM: Growth medium
HE: Hematoxylin-eosin
HGF: Hepatocyte growth factor
IGF: Insulin-like growth factor
MDSC: Muscle-derived stem cell
MHC: Myosin heavy chain
MSC: Mesenchymal stem cell
MuSC: Muscle-specific stem cell
NGF: Nerve growth factor
PGE2: Prostaglandin E2
qPCR: Quantitative real-time polymerase chain reaction
RARγ: Retinoic acid receptor-γ
SC: Satellite cell
SD: Sprague Dawley
VEGF: Vascular endothelial growth factor
VML: Volumetric muscle loss
